# Роль противодиабетических препаратов в терапии болезни Альцгеймера: систематический обзор

**DOI:** 10.14341/probl13183

**Published:** 2023-11-11

**Authors:** А. Н. Ишмуратова, М. А. Абрамов, К. О. Кузнецов, М. В. Иванюта, З. Ф. Шакирова, А. И. Китапова, М. Д. Усмонов, Л. М. Черноусова, Л. И. Валеева, А. Ю. Кузнецова, А. С. Баисламов, А. Р. Шайхетдинова, А. А. Миргалиев, С. Т. Орозбердиев, К. И. Якупова

**Affiliations:** Башкирский государственный медицинский университет; Тульский государственный университет; Башкирский государственный медицинский университет; Российский национальный исследовательский медицинский университет им. Н.И. Пирогова; Башкирский государственный медицинский университет; Башкирский государственный медицинский университет; Башкирский государственный медицинский университет; Ростовский государственный медицинский университет; Башкирский государственный медицинский университет; Башкирский государственный медицинский университет; Башкирский государственный медицинский университет; Башкирский государственный медицинский университет; Башкирский государственный медицинский университет; Башкирский государственный медицинский университет; Башкирский государственный медицинский университет

**Keywords:** сахарный диабет, болезнь Альцгеймера, противодиабетические препараты, метформин, инкретины, тиазолидиндионы, инсулин

## Abstract

Недавние исследования показывают, что болезнь Альцгеймера (БА) имеет множество общих связей с состояниями, ассоциированными с инсулинорезистентностью, включая нейровоспаление, нарушение сигнализации инсулина, окислительный стресс, митохондриальную дисфункцию и метаболический синдром. Авторами был произведен электронный поиск публикаций в базах данных PubMed/MEDLINE и Google Scholar с использованием ключевых слов “amyloid beta”, “Alzheimer type-3-diabetes”, “intranasal insulin”, “metformin”, “type 2 diabetes mellitus”, “incretins” и “PPARγ agonists”. Систематический поиск литературы проводился среди исследований, опубликованных в период с 2005 по 2022 г. Авторами были использованы следующие критерии включения: 1) терапия БА и/или сахарного диабета 2 типа, если ожидаемый результат касался риска снижения когнитивных функций или развития деменции; 2) возраст участников исследования >50 лет; 3) тип исследований, включенных в настоящий обзор, представлял собой рандомизированные клинические испытания, популяционные обсервационные исследования или исследования типа «случай-контроль», проспективные когортные исследования, а также обзоры и метаанализы; 4) включенные статьи были написаны на английском языке. В последние годы наблюдается значительный интерес к выявлению механизмов действия противодиабетических препаратов и их потенциальному применению при БА. Исследования на людях с участием пациентов с легкими когнитивными нарушениями и БА показали, что введение некоторых противодиабетических препаратов, таких как интраназальный инсулин, метформин, инкретины и тиазолидиндионы, может улучшить когнитивные функции и память. Целью настоящего исследования является оценка эффективности противодиабетических препаратов при терапии БА. По результатам исследования метформин, интраназальный инсулин, тиазолидиндионы и инкретины показали положительный эффект как на людях, так и в животных моделях. Последние исследования показывают, что тиазолидиндионы могут активировать пути в головном мозге, которые регулируются инсулиноподобным фактором роста типа 1; однако росиглитазон может представлять значительный риск развития побочных эффектов. Результаты клинических исследований по применению метформина при БА ограничены и противоречивы.

## Abstract (en)

Recent studies show that Alzheimer’s disease (AD) has many common links with conditions associated with insulin resistance, including neuroinflammation, impaired insulin signaling, oxidative stress, mitochondrial dysfunction and metabolic syndrome. The authors conducted an electronic search for publications in the PubMed/MEDLINE and Google Scholar databases using the keywords “amyloid beta”, “Alzheimer type-3-diabetes”, “intranasal insulin”, “metformin”, “type 2 diabetes mellitus”, “incretins” and “PPARy agonists». A systematic literature search was conducted among studies published between 2005 and 2022. The authors used the following inclusion criteria: 1) Subjects who received therapy for AD and/or DM2, if the expected result concerned the risk of cognitive decline or the development of dementia; 2) The age of the study participants is > 50 years; 3) The type of studies included in this review were randomized clinical trials, population-based observational studies or case-control studies, prospective cohort studies, as well as reviews and meta-analyses; 4) The included articles were written in English. In recent years, there has been considerable interest in identifying the mechanisms of action of antidiabetic drugs and their potential use in AD. Human studies involving patients with mild cognitive impairment and Alzheimer’s disease have shown that the administration of certain antidiabetic drugs, such as intranasal insulin, metformin, incretins and thiazolidinediones, can improve cognitive function and memory. The purpose of this study is to evaluate the effectiveness of antidiabetic drugs in the treatment of AD. According to the results of the study, metformin, intranasal insulin, thiazolidinediones and incretins showed a positive effect both in humans and in animal models. Recent studies show that thiazolidinediones can activate pathways in the brain that are regulated by IGF-1; however, rosiglitazone may pose a significant risk of side effects. The results of clinical studies on the use of metformin in AD are limited and contradictory.

## ВВЕДЕНИЕ

Болезнь Альцгеймера (БА) — хроническое нейродегенеративное заболевание, которое является наиболее распространенной причиной развития деменции у пожилых людей [1]. На сегодняшний день в мире деменцией страдают около 47 млн человек, а к 2025 г. прогнозируется увеличение их числа до 131 млн [2]. Самым значимым генетическим фактором риска развития БА является наличие полиморфного аллеля APOE4, а основным морфологическим проявлением заболевания — отложение β-амилоида. По оценкам, 25% населения являются носителями APOE4 [3].

Сахарный диабет (СД) — одно из наиболее распространенных хронических метаболических заболеваний, характеризующееся множественными осложнениями и повышенным риском преждевременной смерти. По состоянию на 2019 г. в мире насчитывается более 463 млн больных СД, а наиболее распространенным является сахарный диабет 2 типа (СД2) [4].

СД2 также имеет корреляцию с повышенным риском развития деменции [5], в частности БА, на 45–90% [6]. Исследование, проведенное в Роттердаме одним из первых, показало повышенный риск развития деменции, ассоциированный с СД2 [7]. Кроме того, было показано, что пациенты с СД2 имеют более высокий риск легкого когнитивного расстройства (ЛКР) [8]. Инсулинорезистентность и гипергликемия как проявления СД2 оказывают пагубное влияние на когнитивные способности, поскольку инсулин и инсулиноподобный фактор роста (ИФР) выполняют важную функцию в обеспечении когнитивных способностей [9].

Недавние исследования показывают, что БА имеет множество общих связей с состояниями, ассоциированными с инсулинорезистентностью, включая нейровоспаление, нарушение сигнализации инсулина, окислительный стресс, митохондриальную дисфункцию и метаболический синдром [10]. Таким образом, БА можно считать метаболическим заболеванием, вызванным резистентностью к инсулину и ИФР в головном мозге, именно поэтому был предложен термин СД 3 типа (СД3) [11]. СД3 — это, по сути, неспособность клеток головного мозга реагировать на инсулин, что приводит к нарушению синаптической функции, метаболизма и иммунного ответа. Взаимосвязь между сигнализацией инсулина и БА или когнитивными нарушениями также может быть доказана результатами исследований, отражающих улучшение когнитивных функций у пациентов с БА на фоне терапии противодиабетическими препаратами, такими как интраназальный инсулин, метформин, тиазолидиндионы и инкретины. Основываясь на исследованиях, которые поддерживают концепцию о том, что БА является метаболическим заболеванием головного мозга [12], и на нарастающих данных общности патогенеза БА и СД2, возникает повышенный интерес к изучению возможности применения противодиабетических препаратов, одобренных для терапии СД2, для лечения БА.

Многочисленные клинические исследования оценивали степень влияния противодиабетических препаратов на патологические проявления БА [13–15], в то время как исследования на животных показали их положительные эффекты при патологии тау-протеина [16] и β-амилоида [17], нейрогенезе [18], оксидативном стрессе [19], нейровоспалении [20], а также они способны улучшать синаптические и когнитивные функции [21]. Целью настоящего исследования является оценка эффективности противодиабетических препаратов при терапии БА.

## МАТЕРИАЛЫ И МЕТОДЫ

Авторами был произведен электронный поиск публикаций в базах данных PubMed/MEDLINE и Google Scholar с использованием ключевых слов “amyloid beta”, “Alzheimer type-3-diabetes”, “intranasal insulin”, “metformin”, “type 2 diabetes mellitus”, “incretins” и “PPARγ agonists”. Систематический поиск литературы проводился среди исследований, опубликованных в период с 2005 по 2022 г.

Авторами были использованы следующие критерии включения: 1) терапия БА и/или СД2, если ожидаемый результат касался риска снижения когнитивных функций или развития деменции; 2) возраст участников исследования >50 лет; 3) тип исследований, включенных в настоящий обзор, представлял собой рандомизированные клинические испытания, популяционные обсервационные исследования или исследования типа «случай-контроль», проспективные когортные исследования, а также обзоры и метаанализы; 4) включенные статьи были написаны на английском языке.

Включенные и исключенные исследования были структурированы в соответствии с предпочтительными элементами отчетности для систематических обзоров и метаанализов (PRISMA) [22]. Всего было выявлено 1644 исследования (Google Scholar — 1162; PubMed — 482). 1387 было исключено при оценке названия и/или резюме (674 — исследование других аспектов патологии; 286 — отсутствовала терапия БА или ЛКР; 42 — нет упоминаний СД2; 77 — нет упоминаний БА; 8 — статьи не на английском языке; 2 — главы книг; 298 — исследования имели низкое методологическое качество). 164 исследования были исключены после их прочтения (21 — популяция не соответствует критериям включения; 2 — копии; 47 — исследование выходило за рамки наших интересов; 19 — минимальное упоминание СД2; 11 — минимальное упоминание БА; 64 — диагноз БА был не подтвержден (МКБ-10, DSM IV)). В конечном итоге было проанализировано 93 исследования (1 — метаанализ; 27 — обзоры; 65 — исследовательские работы). В настоящем обзоре обсуждаются результаты наиболее важных из них.

## РЕЗУЛЬТАТЫ И ОБСУЖДЕНИЕ

Основные характеристики включенных исследований представлены в таблице 1.

**Table:** Таблица 1. Противодиабетические препараты для лечения БА у людей

Исследование	Проводимая терапия	Исследуемая популяция	Результаты
M.A. Reger [14]	Интраназальный инсулин	ЛКР	Улучшение памяти и когнитивных функций
M. Rosenbloom и соавт. [15]	Интраназальный инсулин	БА	Улучшение когнитивных функций у пациентов с отсутствием аллеля APOE4
S. Craft и соавт. [23]	Интраназальный инсулин	БА	Улучшение когнитивных и функциональных способностей
A. Claxton и соавт. [13]	Интраназальный инсулин	БА и ЛКР	Улучшение когнитивной, вербальной и аудиовизуальной памяти
T.P. Ng и соавт. [24]	Метформин	СД2	Снижение риска когнитивных нарушений
C.C. Hsu и соавт. [25]	Метформин	СД2	Снижение риска развития деменции на 24%
A.M. Koenig и соавт. [26]	Метформин	ЛКР	Положительное влияние на исполнительные функции, а также некоторые улучшения памяти и внимания
J.A. Luchsinger и соавт. [27]	Метформин	ЛКР	Значительное улучшение вербальной памяти
E.M. Moore и соавт. [28]	Метформин	БА	Повышенный риск когнитивных нарушений
P. Imfeld и соавт. [29]	Метформин	СД2	Повышенный риск когнитивных нарушений
M. Gejl и соавт. [30]	Лираглутид	БА	Умеренные нейропротективные эффекты, проявляющиеся в улучшении метаболизма глюкозы в головном мозге
M. Gold и соавт. [31]	Росиглитазон	БА	Отсутствие положительного эффекта
G.S. Watson и соавт. [32]	Росиглитазон	БА и ЛКР	Улучшение внимания и замедление запоминания
M.E. Risner и соавт. [33]	Росиглитазон	Пациенты, страдающие БА, не являющиеся носителями аллеля APOE4	Улучшение по шкале оценки БА ADAS-Cog
A.M. Abbatecola и соавт. [34]	Росиглитазон	СД2	Протективное действие в отношении когнитивных нарушений
H. Hanyu и соавт. [35]	Пиоглитазон	БА и СД2	Улучшение когнитивных и метаболических функций
T. Sato и соавт. [36]	Пиоглитазон	БА и СД2	Улучшение когнитивных способностей и мозгового кровотока в теменной доле

## Интраназальный инсулин

Инсулин выполняет множество важных функций в головном мозге, связанных с регулированием потребления пищи, массой тела, пищевыми привычками, а также с энергетическим гомеостазом [37].

Было высказано предположение, что БА может являться метаболическим заболеванием мозга, обусловленным резистентностью к инсулину и ИФР [12].

Некоторые исследования показали, что введение инсулина пациентам с БА снижает действие киназ, которые способствуют гиперфосфорилированию тау-протеина, а также повышают β-амилоидный клиренс и синаптическую пластичность [38]. На самом деле более ранние исследования S. Craft и соавт. показали значительное улучшение памяти у пациентов с БА при гиперинсулинемии, что подтверждает важную роль инсулина в ее регулировании [39]. Следовательно, можно предположить, что увеличение концентрации инсулина в головном мозге может способствовать регрессу некоторых симптомов БА. Однако периферическое введение инсулина несет в себе значительный риск развития гипогликемических состояний, а также возможны трудности при прохождении гематоэнцефалического барьера. Использование интраназального инсулина позволит избежать развития гипогликемии, а также обойти гематоэнцефалический барьер, поскольку через носовые ходы он достигает коры и гиппокампа в течение 15–30 минут [40].

В небольшом исследовании (n=24) оценивалась эффективность недельного введения интраназального инсулина по сравнению с плацебо у пациентов с ЛКР или ранней БА [14]. Авторы отметили улучшение памяти и когнитивных способностей у пациентов, получающих терапию интраназальным инсулином. Кроме того, в исследовании S. Craft и соавт. систематическое введение интраназального инсулина в течение 4 мес у 104 пациентов с ЛКР улучшило когнитивные и функциональные способности, при этом изменения были выражены как в концентрации β-амилоида, так и в соотношении β-амилоида/тау-протеин [23]. Было показано, что инсулин улучшает скорость метаболизма глюкозы в головном мозге [23]. Необходимо отметить, что в этом исследовании интраназальный инсулин оказался эффективным терапевтическим методом для пациентов с БА, без побочных эффектов при длительном применении.

В некоторых клинических исследованиях оценивали инсулин короткого действия, в то время как в других тестировались аналоги инсулина длительного действия. В недавнем исследовании, в котором авторы проводили сравнение эффективности НПХ-инсулина (нейтральный протамин Хагедорна) с инсулином детемир и плацебо у взрослых пациентов с ЛКР или БА, было выявлено, что НПХ-инсулин улучшал память через 2 и 4 мес по сравнению с плацебо, в то время как инсулин детемир не показал никаких существенных эффектов [41]. Кроме того, введение НПХ-инсулина было связано со снижением соотношения тау-P181/β-амилоид; однако различные генетические факторы, такие как наличие APOE4, оказывали влияние на уровень инсулина и резистентность к нему [41].

APOE4 является самым сильным генетическим фактором риска развития БА, около 25% населения являются носителями по крайне мере одного аллеля ε4 [3]. На фоне терапии инсулином происходило улучшение когнитивных функций у пациентов с БА, которые не являлись носителями APOE4, в то время как у носителей APOE4 не было обнаружено никаких улучшений; в некоторых случаях симптомы заболевания ухудшались [14][42]. A. Claxton и соавт. изучали эффекты интраназального введения инсулина детемир пациентам с ЛКР и БА, по результатам исследования было выявлено улучшение когнитивной, вербальной и аудиовизуальной памяти [13]. Значительное улучшение вербальной памяти и инсулинорезистентности у носителей APOE4 наблюдалось после 3 нед терапии, в то время как никаких улучшений у людей без APOE4 не наблюдалось.

В клиническом исследовании II/III фазы, посвященном оценке эффективности интраназального инсулина для улучшения памяти [43], использовались два различных устройства для доставки 20 МЕ инсулина (или плацебо) после завтрака и ужина 240 пациентам с ЛКР или ранней БА. После 1 года терапии не было обнаружено статистически значимого влияния интраназального инсулина на когнитивные способности в основной когорте из 240 пациентов, которые использовали одно из двух устройств. Тем не менее 49 пациентов, использующих другое устройство, продемонстрировали замедление ухудшения состояния по шкале ADAS-COG-12 в течение одного года [43]. Следует отметить, что в этом исследовании изменение устройства доставки инсулина в середине эксперимента, возможно, сыграло важную роль и оказало влияние на результаты.

## Метформин

Метформин — это бигуанид, который увеличивает поглощение глюкозы, подавляет глюконеогенез в печени и повышает чувствительность к инсулину в тканях (рис. 1). Метформин является препаратом выбора для пациентов с СД2, в основном за счет положительного влияния на уровень гликированного гемоглобина (HbA1c), массу тела, сердечно-сосудистые заболевания, а также безопасности его использования [44]. На сегодняшний день данные клинических исследований по использованию метформина при БА ограничены, а результаты неубедительны.

**Figure:**
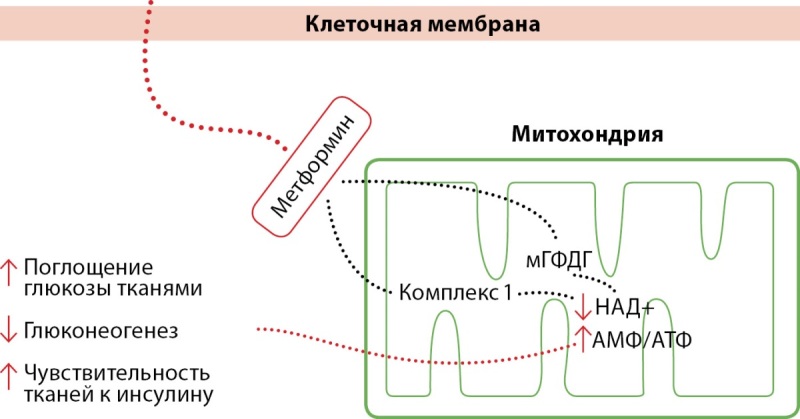
Рисунок 1. Механизм действия метформина.Примечание. Метформин действует в печени, снижая выработку глюкозы в печени, ингибируя глюконеогенез и гликогенолиз. Метформин также действует в мышцах, повышая чувствительность к инсулину, улучшая поглощение периферической глюкозы и замедляя поглощение глюкозы из кишечника. Метформин ингибирует комплекс дыхательной цепи митохондриала 1 и митохондриальную глицеринфосфатдегидрогеназу (мГФДГ), что приводит к снижению уровней НАД+ и АТФ и к вышеуказанным результатам.

Было показано, что метформин может значительно улучшать когнитивные функции у пациентов с СД2 [45]. E.M. Moore и соавт. наблюдали повышенный риск развития когнитивных нарушений у пациентов с СД2 на фоне длительной терапии метформином [28]. Напротив, T.P. Ng и соавт. сообщили, что метформин снижает риск когнитивных нарушений у пациентов с СД2 в возрасте 55 лет и старше, которые наблюдались более 4 лет [24]. В первом исследовании [28], возможно, отрицательные результаты были обусловлены дефицитом витамина B12. Авторы исследования заявляют, что добавки витамина B12 и кальция смягчили вышеупомянутый дефицит и оказали благотворное влияние на когнитивные функции. В исследовании, проведенном системой национального медицинского страхования Тайваня, анализировалась крупная структурированная база данных людей в возрасте 50 лет и старше, у 25 393 был установлен диагноз СД2, а у 101 816 диагноз СД2 отсутствовал [25]. Исследователи установили, что распространенность деменции была выше в 2,6 раза у пациентов с СД2 [25]. В частности, было установлено, что метформин снижает риск развития деменции на 24% по сравнению с пациентами, которые не использовали противодиабетические препараты. В небольшом рандомизированном контролируемом исследовании было обнаружено значимое положительное влияние метформина на исполнительные функции, а также на память и внимание, в то время как уровень гранулоцитарного колониестимулирующего фактора (биомаркер БА) не изменялся [26]. В отличие от приведенных выше исследований, в исследовании типа случай-контроль оценивался риск развития БА на фоне применения различных противодиабетических препаратов в популяции пациентов с СД (n=7086) [29]. Авторы установили, что долгосрочное использование метформина вызывало небольшое увеличение риска развития БА, однако такой эффект отсутствовал при использовании препаратов сульфонилмочевины, тиазолидиндионов, а также инсулина [29]. Возможным объяснением повышенного риска БА и когнитивных нарушений может быть дефицит витамина B12, который часто наблюдается на фоне терапии метформином.

Исходя из вышеизложенного, существует необходимость в дальнейшем изучении роли дефицита витамина B12. Другим важным вопросом является путь введения, поскольку метформин вводится перорально, его действие зависит от способности проходить через гематоэнцефалический барьер, а также от уровня инсулина. Учитывая широкое использование метформина и его влияние на когнитивные функции, необходимы дополнительные исследования, в частности долгосрочное исследование с адекватной выборкой или метаанализ небольших исследований для дальнейшего выяснения механизма его действия.

## Инкретины

Инкретины, включая глюкагоноподобный пептид-1 (ГПП-1) и глюкозозависимый инсулинотропный полипептид (ГИП), являются важными метаболическими пептидами, ответственными за экспрессию гена инсулина, пролиферацию β-клеток и снижение уровня глюкозы путем стимулирования механизмов секреции инсулина (рис. 2) [46].

**Figure:**
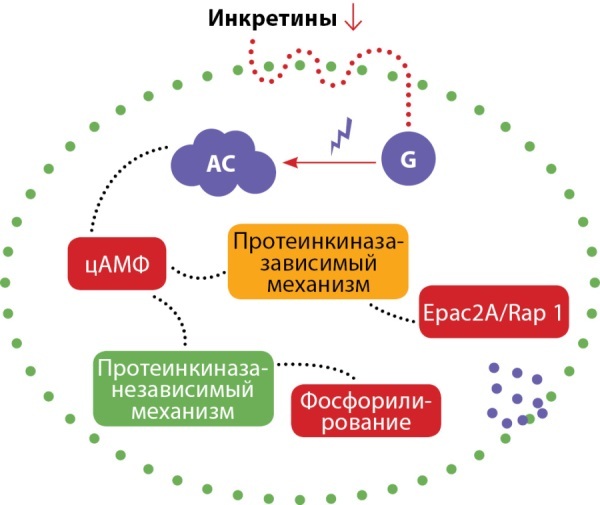
Рисунок 2. Механизм действия инкретинов в β-клетках поджелудочной железы.Примечание. Инкретины индуцируют сигнальный путь циклического аденозинмонофосфата (цАМФ) через рецепторы, связанные с G-белком. Сигнализация цАМФ делится на два разных пути протеинкиназы А: зависимый механизм активирует экзоцитоз инсулина, в то время как независимый регулирует количество гранул инсулина, подготовленных к экзоцитозу.

ГПП-1 выделяется кишечником в ответ на поступление пищи, а его рецепторы (ГПП-1Р), экспрессированные в β-клетках поджелудочной железы, усиливают высвобождение инсулина в ответ на высокий уровень глюкозы. После секреции ГПП-1 фермент дипептидилпептидаза-4 (ДПП-4) разрушает его в течение нескольких минут. Таким образом, аналоги ГПП-1, устойчивые к ДПП-4, были разработаны для клинического применения, а агонисты ГПП-1Р (лираглутид, эксенатид) были одобрены для использования у пациентов с СД [47]. ГПП-1 и его рецепторы встречаются не только в поджелудочной железе и сосудистом эндотелии, но также экспрессируются в головном мозге, в частности в гиппокампе, гипоталамусе, коре головного мозга и в обонятельных луковицах [48]. Инкретины и их аналоги выполняют нейропротекторную функцию [49], поскольку они усиливают пролиферацию клеток, улучшают память и синаптическую пластичность, одновременно уменьшая окислительный стресс, воспаление и β-амилоидные бляшки [50].

Было показано, что лираглутид нормализует распределение рецепторов инсулина в клеточной мембране в модели крыс с БА, тем самым улучшая сигнализацию инсулина [51]. Кроме того, систематическое введение лираглутида трансгенным мышам с БА в течение 8 нед предотвратило основные нейродегенеративные эффекты, наблюдаемые при БА, включая гибель нейронов, нарушение памяти, а также снижение синаптической пластичности в области гиппокампа [52]. В частности, лираглутид снижал осаждение β-амилоидных бляшек на 40–50%, также наблюдалось снижение воспалительного ответа активированных глиальных клеток [52]. У мышей, получавших внутригиппокамапальные инъекции β-амилоида, было отмечено, что предварительное введение лираглутида явилось протективным фактором в отношении развития нарушений пространственной памяти и долговременной потенциации, вызванных β-амилоидом [53]. Дополнительные эксперименты, проведенные на трансгенных мышах, показали, что лираглутид способствует нейрогенезу, положительно влияет на микрососудистую систему головного мозга, а также снижает гиперфосфорилирование тау-протеина при БА [54]. Также лираглутид не только обладает профилактическими свойствами, но и может способствовать обратному развитию ключевых патологических проявлений, которые появляются в заключительной фазе БА в модели мышей [55]. На рисунке 3 представлены терапевтические свойства лираглутида при БА.

**Figure:**
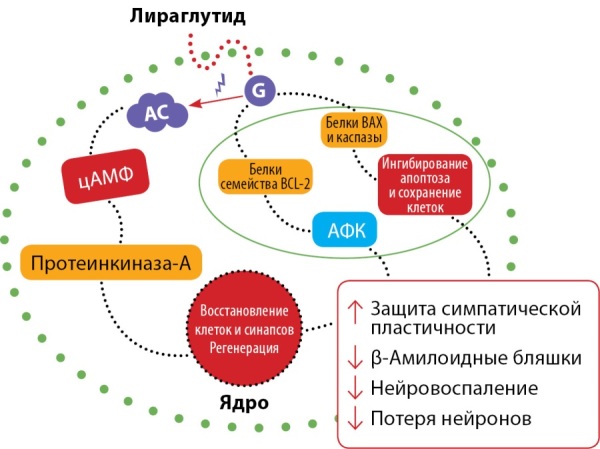
Рисунок 3. Терапевтические свойства лираглутида при БА.

Ингибиторы ДПП-4 также используются в качестве альтернативного метода терапии. Они способны продлить время действия ГПП-1 и ГИП, тем самым контролируя уровень глюкозы при СД2 [56]. E. Kornelius и соавт. обнаружили, что линаглиптин (ингибитор ДПП-4) может восстановить нарушение сигнализации инсулина, индуцированное β-амилоидом в нейронах, что указывает на важную терапевтическую роль, которую ингибиторы ДПП-4 могут играть в нейротоксичности БА [57]. Два других ингибитора ДПП-4, саксаглиптин и вилдаглиптин, показали аналогичную эффективность при пероральном введении трансгенным мышам с БА, что привело к снижению отложения β-амилоида, улучшению памяти и повышению уровня ГПП-1 в гиппокампе, а также к снижению фосфорилирования тау-протеина и маркеров воспаления [58]. Альтернативным веществом является эксенатид, агонист рецепторов ГПП-1 длительного действия, оказывающий нейропротекторное действие при нейродегенеративных заболеваниях, включая БА и болезнь Паркинсона, а также полностью одобрен для использования у пациентов с СД2 [19][50]. Исследование T.R. Bomfim и соавт. показало, что введение эксенатида ингибировало способность β-амилоидных олигомеров ослаблять осевой транспорт [59]. Кроме того, эксенатид улучшает когнитивные функции за счет снижения фосфорилирования серина (субстрат инсулиновых рецепторов в гиппокампе) [53]. Единственное исследование лираглутида, проведенное на пациентах с БА, показало, что 6-месячная терапия привела к развитию умеренного нейропротективного эффекта, что выражалось в улучшении метаболизма глюкозы в головном мозге [29]. В том же исследовании введение лираглутида не повлияло на отложение β-амилоида у пациентов с БА по сравнению с плацебо.

Необходимо проведение дополнительных исследований для уточнения роли инкретинов в терапии БА у людей. Несмотря на многообещающие результаты экспериментов, проведенных на животных, на сегодняшний день отсутствуют данные о возможности регресса БА у людей.

## Тиазолидиндионы (агонисты PPARγ-рецепторов)

У пациентов с СД2 агонисты PPARγ-рецепторов снижают гипергликемию и инсулинорезистентность, а также стабилизируют уровень холестерина (рис. 4).

**Figure:**
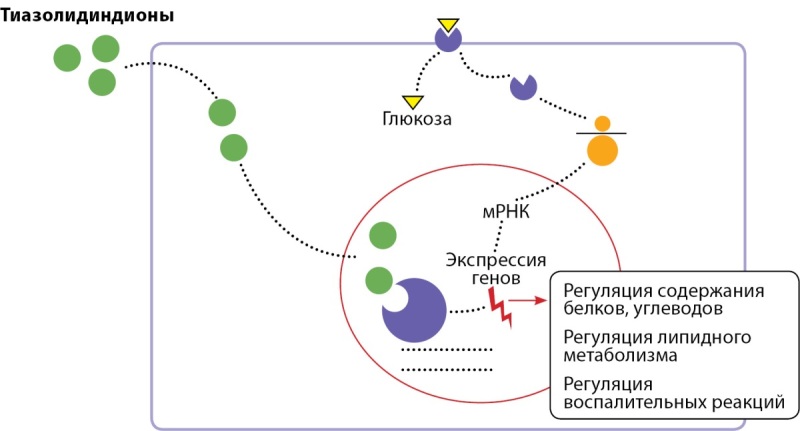
Рисунок 4. Механизм действия тиазолидиндионов.

Наиболее известными агонистами PPARγ являются пиоглитазон и росиглитазон. Возможность их применения у пациентов с БА основана на повышенной экспрессии PPARγ в коре височной доли по сравнению со здоровыми пациентами [60]. PPAR обладают способностью регулировать белковый, углеводный и липидный метаболизм, а также воспалительные реакции, что позволяет использовать их агонисты в качестве терапевтического агента при СД2 и резистентности к инсулину в головном мозге, в то время как последние исследования показывают, что агонисты PPARγ имеют потенциал для активации путей, регулируемых ИФР [61].

Небольшое пилотное исследование, посвященное изучению применения росиглитазона у пациентов с ЛКР и БА, показало, что терапия росиглитазоном в течение 6 мес привела к улучшению внимания и памяти по сравнению с пациентами, принимавшими плацебо [32]. В более крупном исследовании, проведенном вскоре после предыдущего, различные дозы росиглитазона (2,4 или 8 мг) вводились пациентам с легкой и умеренной БА, значительное улучшение по шкале ADAS-Cog наблюдалось после введения 8 мг росиглитазона только у пациентов с отсутствием аллеля APOE4 [33]. APOE4-положительные пациенты не только не показали улучшения, но и, что интересно, продемонстрировали снижение когнитивных функций. Точный механизм, посредством которого APOE4 опосредует действие агонистов PPARγ, на сегодняшний день неизвестен. В другом исследовании оценивался ответ на терапию метформином, росиглитазоном или их комбинацией с целью определения связи между уменьшением резистентности к инсулину и изменением когнитивных функций в течение 36 нед у пациентов с ЛКР и СД2 [34]. Результаты показали, что росиглитазон был более эффективен в отношении нейропротекции у больных СД, чем метформин. Кроме того, T. Sato. и соавт., исследуя пиоглитазон у пациентов с БА и СД2, установили, что введение 15–30 мг пиоглитазона в течение 6 мес улучшало когнитивные способности и мозговой кровоток в теменной доле по сравнению с контрольной группой [36]. В том же исследовании было показано, что введение пиоглитазона снижает уровень инсулина в плазме натощак, что указывает на повышенную чувствительность к инсулину [36]. H. Hanyu и соавт. обнаружили, несмотря на небольшой размер выборки, что пиоглитазон приводил к улучшению по шкале ADAS-Cog, а также метаболической функции у пациентов с БА и СД2 [35]. В другом исследовании того же года оценивалась безопасность пиоглитазона в течение 18-месячного периода у пациентов с БА, но без СД2. Несмотря на хорошую переносимость терапии, результаты исследования не показали эффективности у данной группы пациентов [61].

Что касается клинических исследований, недавний метаанализ, посвященный изучению агонистов PPARγ при БА, включал в общей сложности девять исследований и показал, что только пиоглитазон может обеспечить клиническое улучшение на ранних стадиях БА [62]. В животных моделях агонисты PPARγ снижали экспрессию β-амилоида и оказывали нейропротекторное действие, связанное с гомеостазом кальция в культивируемых нейронах гиппокампа [63].

Соответственно, пиоглитазон в животных моделях БА показал благотворное влияние. В частности, это проявлялось в уменьшении дисфункции мозжечка, сохранении синаптической передачи, улучшении долговременной памяти [64], восстановлении дендритной плотности и нейропластичности [65]. Были также исследования с отрицательными результатами, например M. Gold и соавт. не наблюдали никаких эффектов при использовании росиглитазона у пациентов с легкой и умеренной БА [31]. Кроме того, тиазолидиндионы модулируют сигнализацию Wnt, которая участвует в Aβ-индуцированной нейродегенерации у пациентов с БА [66].

Таким образом, несмотря на доказанные преимущества тиазолидиндионов в терапии БА, наблюдались значительные побочные эффекты, в основном связанные с росиглитазоном, которые проявлялись отеками, инфарктом миокарда и инсультом [66]. Вышеуказанные осложнения, а также отсутствие достаточного количества клинических исследований на сегодняшний день ограничивают их использование и создают необходимость в проведении дополнительных исследований.

## ОБСУЖДЕНИЕ

В настоящем исследовании мы изучили возможность использования антидиабетических препаратов для профилактики и лечения БА. Такие препараты, как метформин, интраназальный инсулин, тиазолидиндионы и инкретины, показали положительный эффект как на людях, так и животных моделях. Последние исследования показывают, что тиазолидиндионы могут активировать пути в головном мозге, которые регулируются ИФР-1; однако росиглитазон может представлять значительный риск развития побочных эффектов. Результаты клинических исследований по применению метформина при БА ограничены и противоречивы, принимая во внимание возможность того, что дефицит витамина B12, часто наблюдаемый при применении метформина, может увеличить когнитивные нарушения и риск развития БА. Метформин также следует рассматривать у отдельных пациентов с преддиабетом в соответствии с критериями Американской диабетической ассоциации. Что касается роли инкретинов и аналогов инкретинов в головном мозге, то можно с уверенностью предположить, что они во многом оказывают нейропротекторное действие. Хотя результаты экспериментов с инкретинами на животных были очень многообещающими, исследования на людях показали противоречивые результаты. Следовательно, роль инкретинов в лечении БА у людей требует дальнейшего изучения. Принимая во внимание, что системное введение инсулина связано с повышенным риском развития гипогликемических состояний, терапевтическое применение инсулина начали изучать как в клинических, так и в доклинических исследованиях. Учитывая его благоприятное воздействие и отсутствие серьезных побочных эффектов, инсулин считается перспективным терапевтическим агентом для лечения БА.

Необходимо проведение дальнейших исследований, посвященных разработке методов лечения БА, в которых необходимо повышать качество отбора пациентов, проводить идентификацию более широкого спектра биомаркеров, которые соответствуют многофакторной природе БА, а также изучать генетические факторы для лучшего понимания взаимодействия генотипа и окружающей среды. АРОЕ4 является сильнейшим генетическим фактором риска БА и важным модулятором интраназального действия инсулина. Дальнейшее понимание жизненно важной роли, которую генотип APOE4 играет в инсулинорезистентности и регуляции, в итоге приведет к разработке более индивидуализированных стратегий лечения пациентов с БА. Следует отметить, что гликемическая вариабельность и преддиабет могут также быть связаны с другими нейродегенеративными заболеваниями с невропатологическими проявлениями, сходными с БА, такими как прогрессирующий надъядерный паралич и кортикобазальный синдром.

Возможные молекулярные механизмы противоальцгеймеровских эффектов препаратов, применяемых для лечения СД, представлены в таблице 2.

**Table:** Таблица 2. Возможные молекулярные механизмы противоальцгеймеровских эффектов препаратов, применяемых для лечения сахарного диабета

Препарат	Молекулярный механизм
Инсулин	Снижение действия киназ, которые способствуют гиперфосфорилированию тау-протеина, а также повышают β-амилоидный клиренс и синаптическую пластичность
Линаглиптин	Восстановление нарушения сигнализации инсулина, индуцированное β-амилоидом в нейронах
Саксаглиптин и вилдаглиптин	Снижение отложения β-амилоида, повышение уровня ГПП-1 в гиппокампе, а также снижение фосфорилирования тау-протеина и маркеров воспаления
Эксенатид	Ингибирование способности β-амилоидных олигомеров ослаблять осевой транспорт
Лираглутид	Защита синаптической пластичности, снижение отложения β-амилоидных бляшек, снижение нейровоспаления и потери нейронов
Пиоглитазон/росиглитазон	Снижение экспрессии β-амилоида и нейропротекторное действие, связанное с гомеостазом кальция в культивируемых нейронах гиппокампа

## ОГРАНИЧЕНИЯ

Это обзорное исследование имеет некоторые ограничения. Из-за неоднородности результатов представленных исследований трудно сделать однозначные выводы о роли противодиабетических препаратов в лечении БА. Дизайн, размер выборки и результаты варьировались между исследованиями. В некоторых исследованиях наблюдались небольшой размер выборки и короткая продолжительность лечения.

## ЗАКЛЮЧЕНИЕ

БА и СД2 — две наиболее глобальные эпидемии последних лет. Противодиабетические средства могут улучшать когнитивные функции, а также нивелировать морфологические проявления заболевания у пациентов с ЛКР и БА. Интраназальный инсулин демонстрирует большие перспективы для лечения БА, и его благоприятная роль модулируется статусом генотипа APOE. Несмотря на обнадеживающие результаты, пока нет достаточных доказательств в поддержку использования противодиабетических препаратов для лечения БА, и необходимы дальнейшие исследования для подтверждения их терапевтического потенциала.

## ДОПОЛНИТЕЛЬНАЯ ИНФОРМАЦИЯ

Источники финансирования. Работа выполнена по инициативе авторов без привлечения финансирования.

Конфликт интересов. Авторы декларируют отсутствие явных и потенциальных конфликтов интересов, связанных с содержанием настоящей статьи.

Участие авторов. Ишмуратова А.Н. — разработка концепции и дизайна исследования, получение и анализ данных, интерпретация результатов; Абрамов М.А. — разработка дизайна исследования, написание статьи; Кузнецов К.О. — анализ данных, написание статьи; Иванюта М.В. — интерпретация результатов, написание статьи; Шакирова З.Ф. — получение и анализ данных, редактирование статьи; Китапова А.И. — интерпретация результатов, редактирование статьи; Усмонов М.Д. — анализ данных, редактирование статьи; Черноусова Л.М. — получение данных, редактирование статьи; Валеева Л.И. — получение данных, редактирование статьи; Кузнецова А.Ю. — получение данных, редактирование статьи; Баисламов А.Е. — получение данных, редактирование статьи; Шайхетдинова А.Р. — получение данных, редактирование статьи; Миргалиев А.А. — получение данных, редактирование статьи; Орзобердиев С.Т. — получение данных, редактирование статьи; Якупова К.И. — получение данных.

Все авторы внесли равный вклад в написание статьи, одобрили финальную версию статьи перед публикацией, выразили согласие нести ответственность за все аспекты работы, подразумевающую надлежащее изучение и решение вопросов, связанных с точностью или добросовестностью любой части работы.
